# Removal of Zn(II) from electroplating effluent using yeast biofilm formed on gravels: batch and column studies

**DOI:** 10.1186/2052-336X-12-8

**Published:** 2014-01-07

**Authors:** Geetanjali Basak, Lakshmi V, Preethy Chandran, Nilanjana Das

**Affiliations:** 1 Environmental Biotechnology Division, School of Bio- Sciences and Technology, VIT University, Vellore, Tamil Nadu, India; 2 School of Chemical and Biotechnology, SASTRA University, Thanjavur, Tamil Nadu, India

**Keywords:** Biofilm on gravels, CLSM, Electroplating effluent, Packed bed column, Yeast, Zn(II) removal

## Abstract

**Background:**

Present study deals with the removal of Zn(II) ions from effluent using yeast biofilm formed on gravels.

**Methods:**

The biofilm forming ability of *Candida rugosa* and *Cryptococcus laurentii* was evaluated using XTT (2,3-bis[2-methoxy-4-nitro-5-sulfophenyl]-2H-tetrazolium-5-carboxanilide) reduction assay and monitored by scanning electron microscopy (SEM), and Confocal laser scanning microscopy (CLSM). Copious amount of extracellular polymeric substances (EPS) produced by yeast species was quantified and characterized by Fourier transform infrared spectroscopy (FT-IR).

**Results:**

Yeast biofilm formed on gravels by *C. rugosa* and C. *laurentii* showed 88% and 74.2% removal of Zn(II) ions respectively in batch mode. In column mode, removal of Zn(II) ions from real effluent was found to be 95.29% by *C. rugosa* biofilm formed on gravels.

**Conclusion:**

The results of the present study showed that there is a scope to develop a cost effective method for the efficient removal of Zn(II) from effluent using gravels coated with yeast biofilm.

## Introduction

Zn (II) is a metal ion which is released into the environment through industrial activities at concentration of physiological and ecological concern. In the Dangerous substances Directive (76/464/EEC) of the European Union, zinc has been registered as List 2 dangerous substance [1]. It is one of the 13 metals found in the contamination list proposed by the United States Environmental Protection Agency (USEPA) [2]. The World Health Organisation (WHO) recommends a 5.0 mg/L maximum acceptable concentration of zinc in drinking water [3]. Zinc is phytotoxic, and the recommended level of zinc for disposal on agricultural land is 2.5 mg/g of dried sludge solids. Effluents from the industries such as manufacture of alloys, sheet metal galvanization, TV picture tubes etc. contain high concentrations of zinc. Discharging these effluents into natural systems adjoining landmasses and sewer systems is a normal practice in small and medium scale industries which poses serious problems to the environment and ecosystems. Therefore, there is a significant need regarding the removal of zinc from effluent [4].

Conventional methods for metal removal include precipitation, filtration, coagulation, evaporation, ion exchange, membrane separation and solvent extraction. However, application of such processes is always expensive and ineffective in terms of energy and chemical products consumption, especially at low concentrations of 1–100 mg/L. Therefore, an alternate cost effective treatment strategy is required which will be eco- friendly. Though biosorption has been regarded as a cost effective technique for removal of Zn(II) ion using microorganisms such as bacteria [5-7], fungi [8,9] and yeast [10-12], application of biofilm may be a better choice for Zn(II) removal from effluent.

Biofilm is a kind of immobilization of microorganisms in a solid matrix and can be applied for bioremediation of effluents. During the last few decades, biofilm reactors have become a focus of interests for the researchers in the field of bioremediation of pollutants. There are reports on the application of bacterial biofilm for zinc removal using granular activated carbon [13,14], moving bed sand filter [15] and combined AS- biofilm process [16]. Reports are scanty regarding the use of yeast biofilm for Zn(II) removal.

Biofilm formation in microorganisms is closely linked with production of extracellular polymeric substance (EPS) which acts as a glue, helping the attachment of cell surface to the submerged structures [17]. Extracellular polymeric substance are mainly composed of polysaccharides, proteins, humic substances and uronic acid [18] which contains several functional groups like carboxyl, phosphoric, amine and hydroxyl groups. EPS has various functions *viz.* induction of cell aggregation [19], producing a protective barrier for cell against harmful products and allowing sorption of inorganic ions from the environment [20]. There is report on the production of a water soluble 300kDa extracellular polymeric substance produced by yeast *Candida albicans* made up of glucose, mannose, ramnose and N-acetyl glucosamine using gas chromatography, gel permeation chromatography, FTIR spectrophotometer, ^1^H and ^13^C NMR spectrophotometer [21].

For large scale effluent treatment, continuous flow operations in column mode are more useful than batch mode. However, little effort has been focused on column study using yeast biofilm for removal of Zn(II) ion from synthetic solutions.

The aim of the present study was (i) to study the biofilm forming ability of yeast isolates and monitoring of biofilm formation through microscopic analysis viz. SEM and CLSM (ii) to quantify the amount of extracellular polymeric substances (EPS) produced by yeast during biofilm formation and characterization of EPS through FT-IR analysis and (iii) to evaluate the process of Zn(II) removal by yeast biofilm in batch mode and to study the removal of Zn(II) ion from real effluent in continuous flow column reactor packed with, gravels coated with yeast biofilm.

## Materials and methods

### Preparation of Zn(II) solution

Zn(II) stock solution (1000 mg/L) was prepared by dissolving 4.55 g of powdered Zn(NO_3_)_2_.6H_2_O (Hi Media, Mumbai, India) in 1000 ml of deionised water. The working solutions of metal were prepared by diluting the stock solution to desired concentrations.

### Yeast and growth condition

Two yeast species were isolated from Common Effluent Treatment Plant (CETP), Ranipet, Vellore, Tamilnadu, India. The yeasts were phenotypically characterized and identified to species levels as *C. rugosa* and *C. laurentii* by Vitek 2 Compact Yeast card reader with software version V2C 03.01 from Council for Food Research and Development (CFRD), Kerela, India. The isolates were subcultured in YEPD (yeast extract: 10 g/L; peptone: 20 g/L; dextrose: 20 g/L) agar slant and maintained at 4°C. Sugarcane bagasse extract having 24 g/L total sugar (pH 5.0) was used as media for the biofilm formation and Zn(II) removal studies.

### Analysis of effluent

Effluent was collected from Krishna electroplating works, located at Kolkata, West Bengal, India. The physico-chemical characteristics of effluent were analyzed promptly after collection using standard analytical methods [22]. The concentration of zinc, copper, nickel and cadmium present in effluent was analyzed using Atomic Absorption Spectrophotometer (Varian AA-240, Australia).

### Biofilm formation

The gravels were collected from a local nursery at VIT University. The size of the gravels was made uniform at a size of 7.5 mm by passing through the mesh. They were dipped in 1 ml culture with 5 × 10^8^ CFU/ml of 48 h grown yeast species, placed for 90 min of adhesion phase at 28°C and were then washed with sterilized phosphate buffered saline to remove loosely adherent cells. One millilitre of sterilized sugarcane bagasse medium was added to the washed pieces and incubated at 28°C for 48 h. The biofilm thus formed was then quantified using XTT reduction assay.

### XTT reduction assay

XTT (sigma, St Louis, MO, USA) solution (1 mg/ml in PBS) was prepared, filters sterilized through a 0.22 μm-pore size filter and stored at -70°C. Menadione (sigma) solution (0.4 mM) was prepared and filter sterilized immediately before each assay. Prior to each assay, XTT solution was thawed and mixed with the menadione solution at a ratio of 5 to 1 by volume. The biofilms formed on gravels were first washed five times using 1ml of PBS, and then 1 ml of PBS and 60 μl of XTT-menadione solution were added to each of the prewashed and control tubes. The tubes were then incubated in the dark for 2 h at 28°C. Following incubation, the colour change in the solution was measured spectrophotometrically at 492 nm (Hitachi U-2800) [21].

### Morphological characterization of yeast biofilm

Yeast biofilm formed on gravels were morphologically characterized using Scanning electron microscopy (Stereo Scan LEO, Model -400*)* following the method of Hawser and Doughlas [23] and Confocal laser scanning microscopy (Olympus FV 1000, America) following the method of Sundar et al. [24].

### Recovery and characterization of Extracellular polymeric substance

Extracellular polymeric substance (EPS) was isolated from culture supernatants of yeast species using acetone precipitation technique [25]. The carbohydrate composition of EPS was also determined using HPLC (chromatograph Waters, Milford, MA, USA) following the method of Simova et al. [26]. The dialysed EPS was characterized through infrared analysis using FT-IR spectrophotometer (Perkin Elmer Spectrum 1).

### Batch studies on Zn(II) removal using yeast biofilm formed on gravels

Yeast biofilm formed on gravels were washed with phosphate buffer to remove the loosely attached cells. The washed gravels were then used for batch studies. In order to study the effect of growth supportive media on Zn(II) removal by yeast biofilm, the batch experiments were conducted by immersing the washed gravels in Zn(II) solution with and without sterilized sugarcane bagasse extract at pH 5.0. The temperature was maintained at 28°C. To study the effects of pH, temperature and initial Zn(II) concentration on Zn(II) removal, experiments were conducted at different pH ranging from 2–8, temperature ranging from 20–40°C and initial Zn(II) concentration ranging from 10–100 mg/L. The dry weight of the biofilm biomass (including yeast cells and EPS) was measured at regular intervals of time. The preweighed gravels with attached biofilm were removed from experimental flasks, carefully blotted to remove excess medium without disrupting the biofilm, dried and weighed. Liquid samples were withdrawn at regular intervals and centrifuged at 10,000 rpm for 5 min. Supernatants were subjected to AAS analysis for residual Zn(II) concentration. All experiments were performed in triplicates and the data presented are mean of triplicates. The Zn(II) removal percentage using yeast biofilm was calculated from the following equation:

(1) $$\frac{C_{i}-C_{f}}{C_{i}}\times 100$$

Where C_*i*_ is the initial concentration of Zn(II) ion (mg/L). C_*f*_ is the final concentration of Zn(II) ion (mg/L).

### Removal of Zn(II) from electroplating effluent in column mode using yeast biofilm

A glass column with an internal diameter of 3 cm and height 15 cm was employed in the column experiments. The column was packed with gravels coated with yeast biofilm. Effluent collected from electroplating industry containing 85 mg/L Zn(II) ions was used in this experiment. Before passing through the column, effluent was mixed with sugarcane bagasse extract, pH was adjusted to 6.0 and fed through the column at a desired flow rate using a peristaltic pump. To study the effect of bed height on Zn(II) removal in column mode, experiments were conducted at three different bed heights *viz.* 4, 8 and 12 cm. The effect of flow rate on Zn(II) ion removal was studied at three different flow rates *viz.* 1, 3 and 5 ml/min. Samples collected from the exit of the column at different time intervals were analyzed by AAS. Effluent was passed through the column till the values reached the US EPA standard (5.0 mg/L).

## Results and discussion

### Yeast biofilm formation

**Figure 1:**
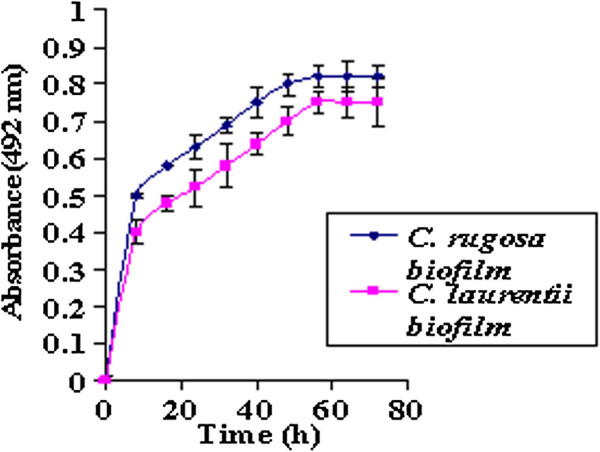
Yeast biofilm formation on gravels determined by XTT reduction assay.

The adherence and subsequent biofilm formation by the yeast species *viz. C. rugosa* and *C. laurentii* on gravels over 72 h were studied using XTT reduction assay. The production of the soluble coloured formazan salt from biofilm forming cells on gravels was a direct reflection of cellular metabolic activity which increased with time (Figure 1). It was evident that the yeast species were capable of biofilm formation.

### Morphological characterization

**Figure 2:**
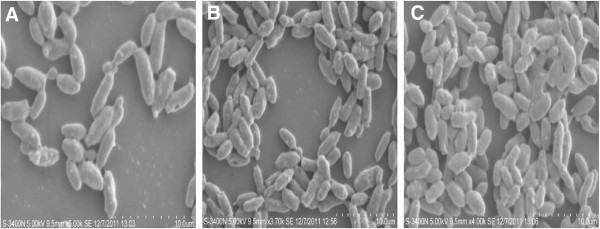
**SEM images showing sequential biofilm formation by *C. rugosa *** **on PVC strips during: (A) 12 h; (B) 24 h and (C) 48 h.**

**Figure 3:**
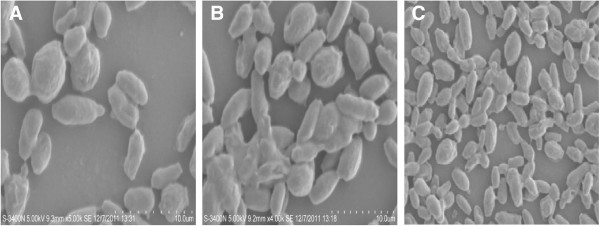
**SEM images showing sequential biofilm formation by *C. laurentii *** **on PVC strips during: (A) 12 h (B) 24 h and (C) 48 h.**

Surface morphology of the biofilms was studied by SEM images. In the initial adherence phase (12 h), the yeast species started colonization on gravels and as the biofilms matured, aggregation complexity increased with time (Figure 2A-C) and (Figure 3A-C).

**Figure 4:**
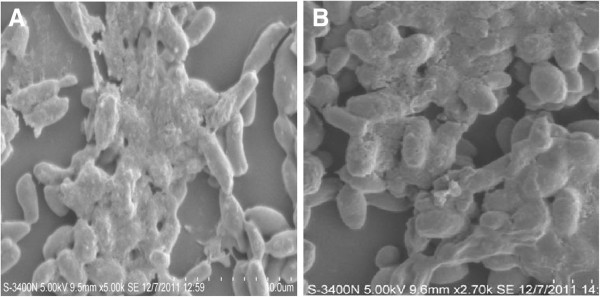
**SEM images showing exopolysaccharide (EPS) production by biofilm forming: (A) *C. rugosa and *** **(B) *C. laurentii.***

Exopolysaccharide production by yeast species was also noted through SEM images. The physical appearance of a developing biofilm as a net like structure consisting of yeast strung together and bound by polysaccharide coating were observed in case of *C. rugosa* (Figure 4A) and *C. laurentii* (Figure 4B). This coating provided sites for the attraction of positively charged metal ions allowing it to act as an ion-exchange medium [13].

**Figure 5:**
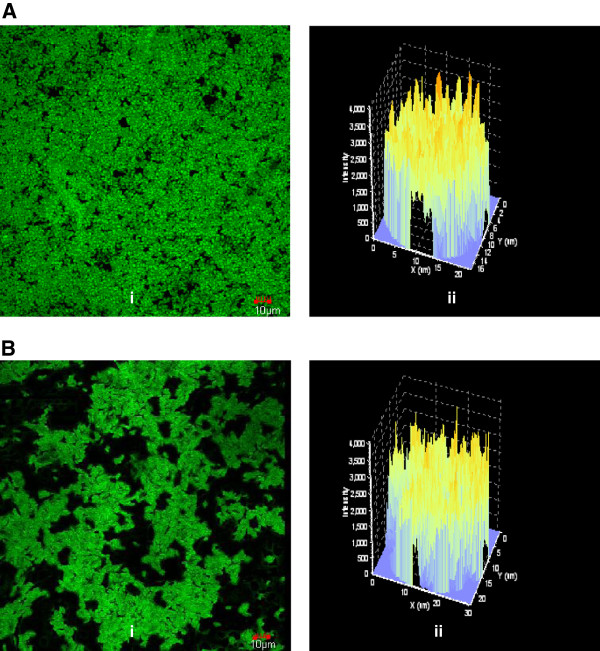
**CLSM imaging of (A) uninteracted and Zn(II) (B) interacted *C. rugosa *** **biofilm after 72 h of growth.**

**Figure 6:**
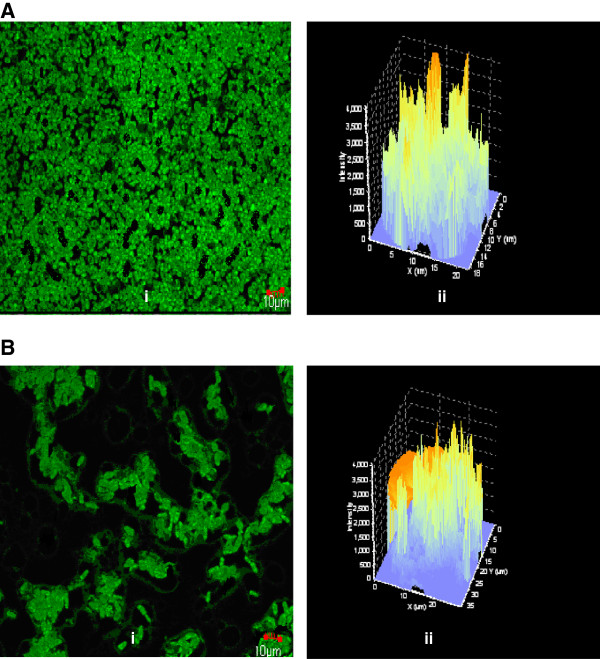
**CLSM imaging of (A) uninteracted and Zn(II) (B) interacted *C. laurentii *** **biofilm after 72 h of growth.**

Formation of yeast biofilms were analysed by CLSM. The uninteracted biofilms on gravels showed more thickness of biomass than the thickness of Zn(II) interacted biofilms. Figure 5A(i) and Figure 6A(i) showed average projections of CLSM z-series images from uninteracted biofilms formed by *C. rugosa* and *C. laurentii* respectively. Figure 5A(ii) and Figure 6A(ii) showed the thickness of biofilm with z-stacks three-dimensional (3D) reconstruction respectively for *C. rugosa* and *C. laurentii*. Thickness of the uninteracted biofilms was calculated as 51.06 ± 1.25 μm for *C. rugosa* and 45.03 ± 2.37 μm for *C. laurentii*. The thickness of biofilm decreased to 37.60 ± 2.89 μm for *C. rugosa* (Figure 5B(i) and Figure 5B(ii)) whereas in case of *C. laurentii* the thickness of biofilm was reduced to 34.78 ± 3.54 μm (Figure 6B(i) and Figure 6B(ii)) after Zn(II) interaction for 12 h.

### Recovery of extracellular polymeric substance (EPS)

**Figure 7:**
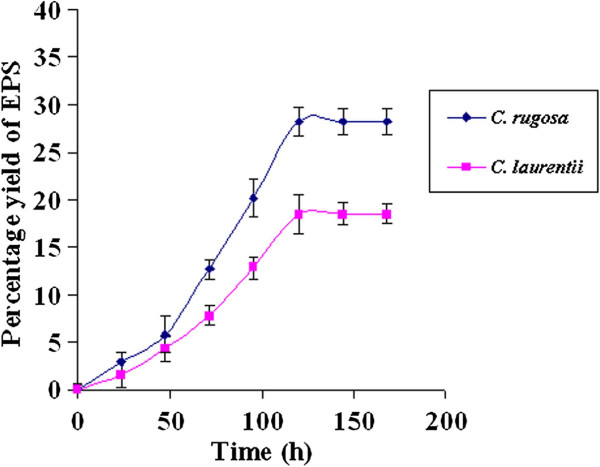
**Time course of exopolysaccharide synthesis.** (temperature: 28°C; time: 168 h).

EPS was recovered by acetone precipitation technique. Growth for 7 days in 1 litre medium, an EPS yield of 28.2% by *C. rugosa* and 18.5% by *C. laurentii* was obtained by precipitation of culture supernatants with acetone. Figure 7 showed the percentage yield of EPS produced by the yeast species at different time intervals. EPS formation protects the yeast cells from environmental chemical toxicity [27].

### Characterization of EPS

**Figure 8:**
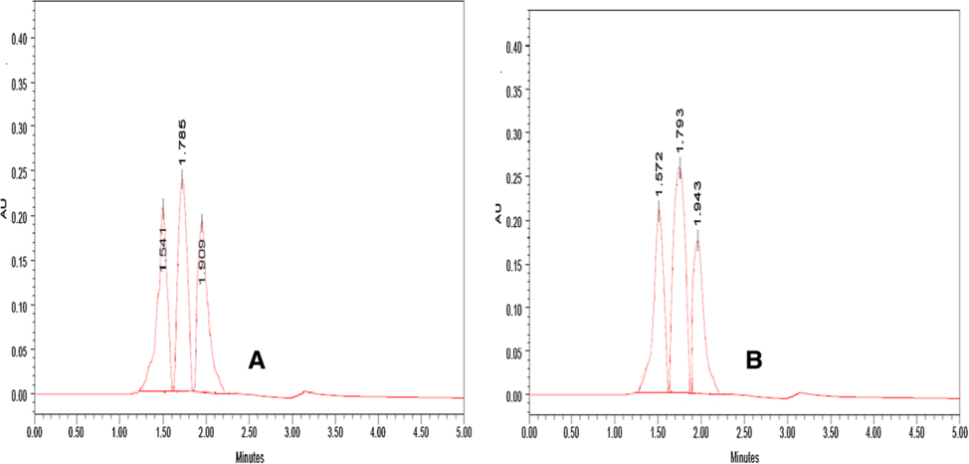
**HPLC analysis of the monosaccharide components of EPS produced by (A) *C. rugosa *** **and (B) *C. laurentii.***

The sugar composition of EPS produced by *C. rugosa* and *C. laurentii* were analysed using HPLC (water 2487 model). The HPLC chromatogram (Figure 8A) showed the presence of three individual sugars *viz.*, glucose (25.59%), mannose (36.14%) and glucuronic acid (17.79%) at 1.541, 1.785 and 1.909 retention times, respectively for EPS produced by *C. rugosa*. Figure 8(B) showed the HPLC peaks which revealed the presence of glucose (18.5%), mannose (33.35%) and glucuronic acid (16.12%) at 1.572, 1.793 and 1.943 retention times, respectively in case of *C. laurentii*. Therefore, the present results indicated that glucose, mannose and glucuronic acids were the most abundant sugars in EPS produced by *C. rugosa* and *C. laurentii.* Similar work was reported in case of yeasts belonging to *Candida, Cryptococcus, Rhodotorula* and *Sporobolomyces* genera which produced EPS such as mannan, glucomannan and glucan [26,28-30]. Since the EPS produced by yeast are more easily separated from the culture broth than those produced by bacteria; they are more attractive for a large scale production [31].

### FT-IR analysis of EPS

**Figure 9:**
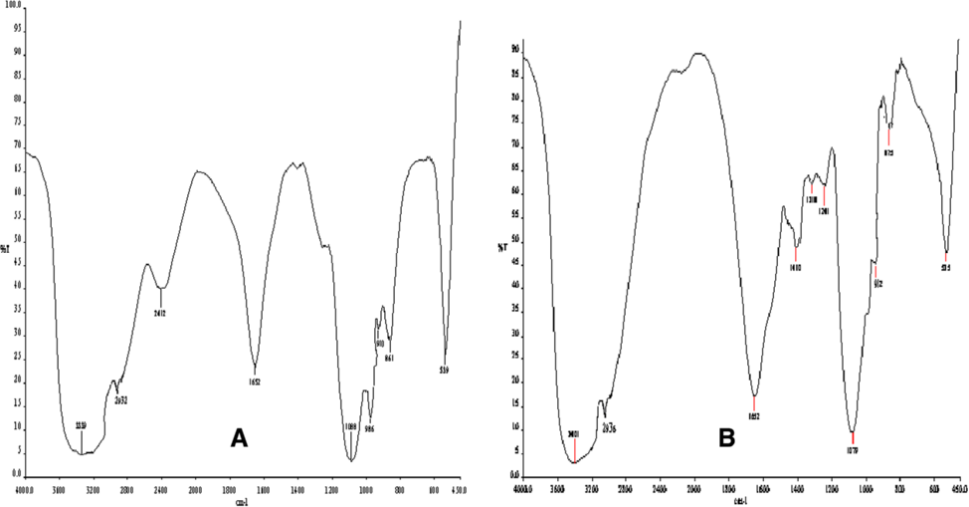
**FT-IR spectra of the exopolysaccharides produced by yeast biofilm (A) *C. rugosa *** **and (B) *C. laurentii*.**

FTIR spectroscopy opens up new possibilities for the fine structural analysis of polysaccharides and its derivatives. In order to investigate the functional groups of the purified EPS, the FT-IR spectra were measured in KBr pellets. Typical IR spectra for the EPS produced by two yeast species *C. rugosa* and *C. laurentii* are presented in Figure 9(A) and Figure 9(B) respectively. The broad and the strong stretching bonds at 3339 cm^-1^ (*C. rugosa*) and 3401 cm^-1^ (*C. laurentii*) indicated bounded hydroxyl (-OH) group.

The absorption bands at 2932 cm^-1^ (*C. rugosa*) and 2936 cm^-1^ (*C. laurentii*) were intensified and assigned to the stretching vibration of the methylene group (C-H). Furthermore, a continuous absorption beginning at approximately in the region of 3339 cm^-1^ for *C. rugosa* and 3401 cm^-1^ for *C. laurentii* are the characteristic of a carbohydrate ring. In comparison with the IR spectra of polysaccharides documented in literature, a characteristic absorption band appeared at 1652 cm^-1^ for the exopolysaccharide produced by both the yeast species. The absorption band at 1652 cm^-1^ were assigned to the stretching vibration of the carboxyl group (C = O). The peaks observed at 1410 cm^-1^ and 1318 cm^-1^ for *C. laurentii* is the symmetric stretching vibrations of carboxylic group (-COO^-^). A broad stretching of C-O-C, C-O at 1000–1200 cm^-1^ indicated the presence of carbohydrates [32]. Specifically, the absorption band appeared at 1088 cm^-1^ (*C. rugosa*) and 1079 cm^-1^ (*C. laurentii*) ascertains the presence of uronic acid, Ο-acetyl ester linkage bonds. The presence of acidic sugars in the EPS may be important, considering the heavy metal-binding properties of the polymers [33]. In addition, the absorption bands in the region 900-800 cm^-1^ were associated to the glycosidic linkage types in polysaccharides. The absorption peaks at 910 cm^-1^ and 861 cm^-1^ in the EPS produced by *C. rugosa* and absorption peaks at 912 cm^-1^and 875 cm^-1^ in *C. laurentii* exopolysaccharide revealed the coexistence of α and β glycosidic bonds [34]. Similar FT-IR results were reported by Ma et al. [35].

### Batch studies on Zn(II) removal using yeast biofilm formed on gravels

Removal of Zn(II) ion using biofilm formed by *C. rugosa* and *C. laurentii* was tested in growth restricted (without sugarcane bagasse extract) and growth supportive (with sugarcane bagasse extract) media at pH 5 and temperature 28°C for 12 h. It was found that Zn(II) removal efficiency by yeast biofilm in growth supportive media was more compared to growth restricted media. In the growth supportive medium, Zn removal was 35% and 29% by *C. rugosa* and *C. laurentii* biofilms respectively, whereas in growth restricted medium 25% and 21% Zn(II) ions was removed by *C. rugosa* and *C. laurentii* biofilms respectively. Therefore, further experiments were carried out with growth supportive media. In the growth supportive media, the doubling times of *C. rugosa* and *C. laurentii* were 90 min and 120 min respectively. Yeast is simple, economical, and rapid, with a doubling time in rich medium of approx 90 min to 140 mins. In addition to sugars, the aqueous extract of sugarcane bagasse also contained nitrogen compounds and other nutrients including sulphates, chlorides, phosphates, potassium, sodium, calcium, iron and copper [36] that enhanced the doubling time of the yeast species. The specific growth rate of *C. rugosa* and *C. laurentii* were found to be 0.135 h^-1^ and 0.129 h^-1^ respectively.

**Figure 10:**
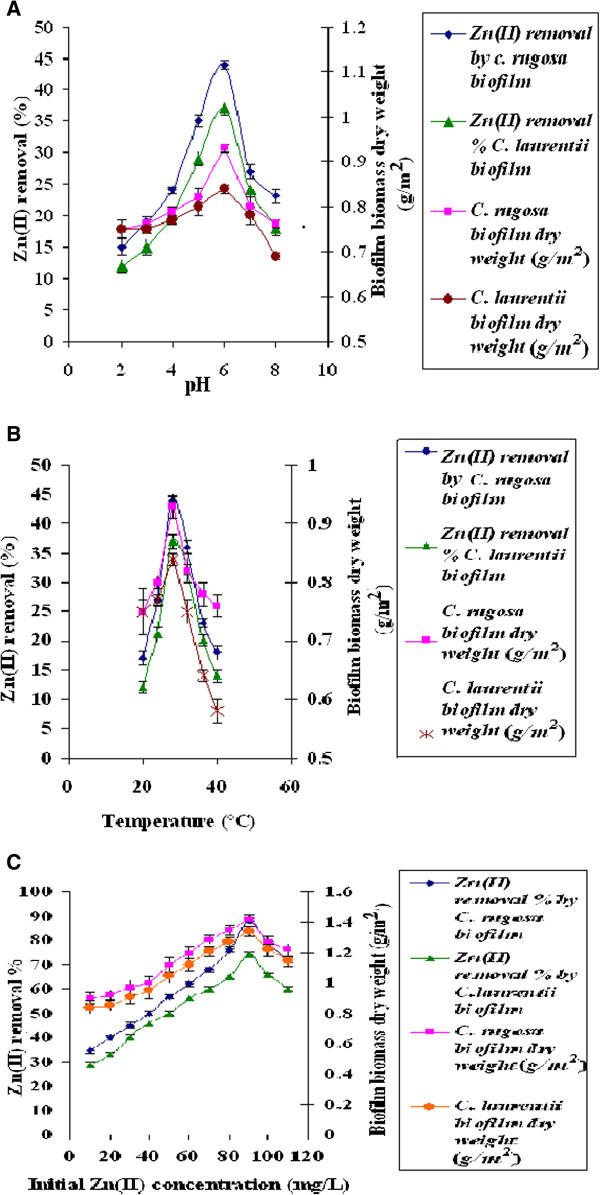
**Effect of parameters on Zn(II) removal by yeast biofilm grown on sugarcane bagasse extract medium. (A)** pH (temperature: 28°C; contact time: 12 h) **(B)** temperature (contact time: 12 h; pH: 6) and **(C)** initial Zn(II) concentration (temperature: 28°C; contact time: 12 h; pH: 6.0).

The effect of initial pH on Zn(II) removal and biofilm biomass was studied. The initial pH significantly affected the growth and Zn(II) removal properties of yeast biofilms. The Zn(II) removal efficiency and biofilm biomass increased when pH increased from 2–6 (Figure 10A). When pH is increased, the negatively charged ligands on the biofilm surface was exposed, resulting in the greater attraction for the cationic Zn(II) ion [37]. pH 6.0 was chosen as optimum for the removal of Zn(II) ions by the yeast biofilm biomass. At pH 6.0, the main chemical groups *viz.* carboxyl, phosphate, sulfydryl, hydroxyl and nitrogen-containing groups of biofilm biomass surface might have participated in the removal of Zn(II) ion [38]. At optimum pH 6.0, *C. rugosa* biofilm showed maximum Zn(II) removal of 44% and biofim biomass dry wt. 0.93 g/m^2^, whereas *C. laurentii* biofilm showed 37% Zn(II) removal and biofilm biomass dry wt was 0.84 g/m^2^ at 12 h.

The temperature of sorption medium is important for energy dependent mechanisms in metal sorption by microorganisms [39]. The optimum temperature for Zn(II) removal by yeast biofilm biomass was found to be 28°C at 12 h. (Figure 10B). At low temperature, the yeast biofilm biomass dry weight and Zn(II) removal was found to be less because the binding of Zn(II) onto yeast biofilm biomass took place only by passive uptake. Biofilm biomass dry weight and Zn(II) removal was found to be minimum at higher temperature. Higher temperatures inhibited the yeast growth due to reduced enzymatic activity [40].

Experiments were performed to study the effects of initial metal concentration on Zn(II) removal by yeast biofilm. Zinc removal efficiency was increased with increase in initial Zn(II)concentration ranging from 10 mg/L to 90 mg/L (Figure 10C). Maximum removal of Zn(II) ion by yeast biofilm occurred at 90 mg/L. The increase in initial Zn concentration increased the driving force to overcome mass transfer resistance of metal ion between aqueous and solid phases [41]. In this study, Zn(II) removal efficiency of *C. rugosa* and *C. laurentii* biofilm was found to be 88% and 74.2% of 90 mg/L respectively. The maximum biofilm biomass (dry wt) produced by *C. rugosa* was 1.42 g/m^2^ and *C. laurentii* was 1.34 g/m^2^ at 12 h.

### Treatment of effluent in column mode using yeast biofilm formed on gravels

**Figure 11:**
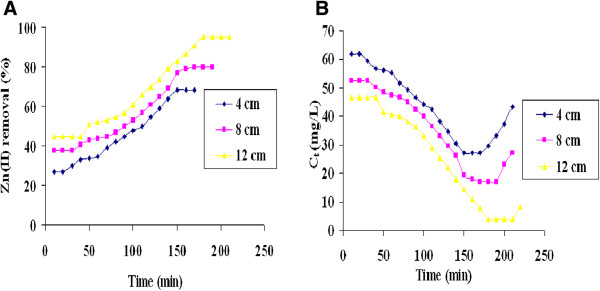
**Removal of Zn(II) from industrial effluent by *C.rugosa *** **biofilm at different bed heights (A) Zn(II) removal percentage; (B) Breakthrough curve (Inlet Zn(II) concentration in the industrial effluent: 85 mg/L; flow rate 1 mL/min; pH; 6.0).**

The *C. rugosa* biofilm formed on gravels was employed for the treatment of effluent in a continuous column mode. Figure 11A and Figure 11B showed the removal percentage and breakthrough curves of Zn(II) ion removal respectively at different bed heights. The removal percentage of Zn(II) ion increased from 68.12% to 95.29% with the increase in bed height from 4 cm to 12 cm at the flow rate of 1 mL/ min (Figure 11A). The breakthrough time (t_b_) and the exhaustion time (t_e_) increased with the increase in bed height (Figure 11B). High percentage of Zn(II) removal was noted at maximum bed height 12 cm.

**Figure 12:**
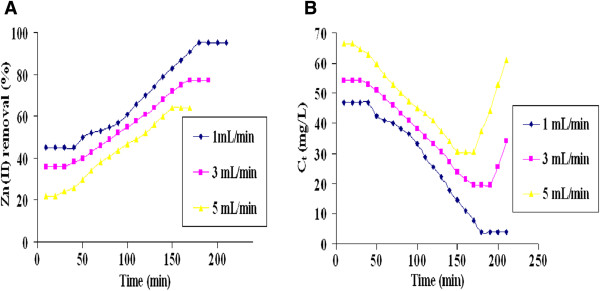
**Removal of Zn(II) from industrial effluent by *C. rugosa *** **biofilm at different flow rates (A) Zn(II) removal percentage (B) Breakthrough curve (Inlet Zn(II) concentration in the industrial effluent: 85 mg/L; bed height: 12 cm; pH; 6.0).**

The effect of flow rate on Zn(II) removal was also evaluated using three different flow rates (1, 3 and 5 mL/ min) at optimum bed height of 12 cm. Figure 12A depicted that as the flow rate decreased from 5 to 1 mL/min, the Zn(II) removal percentage increased from 64 to 95.29%. Figure 12B showed that an increase in flow rate resulted decrease in breakthrough and exhaustion times due to insufficient residence time of the metal ions in the column [42]. Therefore, the maximum Zn(II) removal of 95.29% was obtained in the column mode at the highest bed height (12 cm) and the lowest flow rate (1 mL/min). The total residence time was 180 minutes and the biofilm biomass dry weight was found to be 2.98 g/m^2^ for the Zn(II) removal upto 95.29%, leaving 4 mg/L residual Zn(II) ion in the treated effluent. The residual concentration of Zn(II) in the treated effluent was less than the US EPA standard (5.0 mg L^-1^). The physicochemical characteristics of the effluent before and after treatment in the column are shown in the Table 1. There was significant difference in the physicochemical properties of industrial effluent after biofilm treatment. The residual concentrations of other metals *viz.* Ni(II), Cd(II) and Cu(II) were found to be 2.8 mg/L, 1.8 mg/L and 2.6 mg/L respectively. These residual concentrations were less than the permissible limits of each metal.

**Table 1:** Physico-chemical analysis of electroplating effluent

**Parameters**	**Before treatment**	**After treatment**
**pH**	7.63 ± 0.17	6.4 ± 0.63
**Conductivity (μΏ)**	5.68 ± 0.20	2.1 ± 0.02
**TSS (mg/L)**	1310 ± 3.7	615 ± 5.2
**TDS (mg/L)**	1137 ± 4.1	550 ± 4.5
**COD (mg/L)**	61 ± 1.2	23 ± 0.64
**Zn(II) (mg/L)**	85 ± 0.64	4 ± 0.4
**Cd(II) (mg/L)**	10 ± 0.54	1.8 ± 0.09
**Ni(II) (mg/L)**	21 ± 0.76	2.8 ± 0.17
**Cu(II) (mg/L)**	20 ± 0.43	2.6 ± 0.15

## Conclusions

The present study showed the capability of the yeast species to form biofilm onto natural substrate like gravels. The EPS characterization study showed that extrapolymeric substances produced by the yeast species are made up of glucose, mannose and glucuronic acid subunits which would protect the cells from environmental chemical toxicity. This study gives an insight about the ability of artificially formed yeast biofilms on gravels to remove Zn(II) ions from aqueous solution as an inexpensive and alternative method to traditional techniques for removal of Zn(II) from waste waters. The potentiality of *C. rugosa* biofilm for removal of Zn(II) ions from real effluent was also studied in column mode.

## Competing interests

The authors declare that they have no competing interest.

## Authors’ contribution

GB, LV and ND participated in the design of the study and draft the manuscript. GB carried out the experimental studies. PC helped for the instrumental analyses. ND corrected the manuscript. All the authors finally approved the manuscript.
